# Novel Scaffolds for Modulation of NOD2 Identified by Pharmacophore-Based Virtual Screening

**DOI:** 10.3390/biom12081054

**Published:** 2022-07-29

**Authors:** Samo Guzelj, Tihomir Tomašič, Žiga Jakopin

**Affiliations:** Faculty of Pharmacy, University of Ljubljana, Aškerčeva cesta 7, 1000 Ljubljana, Slovenia; samo.guzelj@ffa.uni-lj.si (S.G.); tihomir.tomasic@ffa.uni-lj.si (T.T.)

**Keywords:** antagonist, homology modeling, NOD2, pharmacophore modeling, virtual screening

## Abstract

Nucleotide-binding oligomerization domain-containing protein 2 (NOD2) is an innate immune pattern recognition receptor responsible for the recognition of bacterial peptidoglycan fragments. Given its central role in the formation of innate and adaptive immune responses, NOD2 represents a valuable target for modulation with agonists and antagonists. A major challenge in the discovery of novel small-molecule NOD2 modulators is the lack of a co-crystallized complex with a ligand, which has limited previous progress to ligand-based design approaches and high-throughput screening campaigns. To that end, a hybrid docking and pharmacophore modeling approach was used to identify key interactions between NOD2 ligands and residues in the putative ligand-binding site. Following docking of previously reported NOD2 ligands to a homology model of human NOD2, a structure-based pharmacophore model was created and used to virtually screen a library of commercially available compounds. Two compounds, **1** and **3**, identified as hits by the pharmacophore model, exhibited NOD2 antagonist activity and are the first small-molecule NOD2 modulators identified by virtual screening to date. The newly identified NOD2 antagonist scaffolds represent valuable starting points for further optimization.

## 1. Introduction

**Figure 1:**
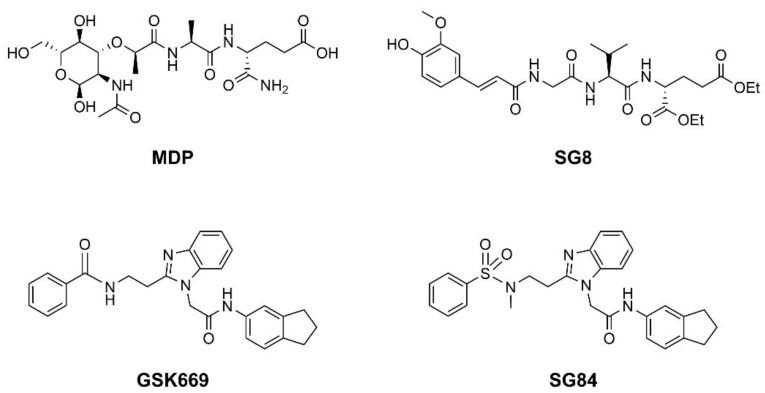
Representative NOD2 agonists MDP and SG8, and NOD2 antagonists GSK669 and SG84.

Nucleotide-binding oligomerization domain-containing protein 2 (NOD2) is an intracellular innate immune receptor that belongs to the pattern recognition receptor (PRR) superfamily [1]. These receptors act as immune sentinels, orchestrating the first line of defense against invading pathogens by recognizing and responding to conserved pathogen-associated molecular patterns (PAMPs), such as nucleic acids and bacterial cell wall components [2]. NOD2, which is expressed in both hematopoietic (T cells, B cells, macrophages, dendritic cells) and some non-hematopoietic cells (Paneth cells, goblet cells, enterocytes), responds primarily to “muropeptides”, specific substructures of bacterial peptidoglycan, with muramyl dipeptide (MDP; Figure 1) considered as the smallest fragment of peptidoglycan still capable of activating NOD2 [3,4,5].

NOD2 consists of two effector N-terminal caspase recruitment domains (CARDs), a central nucleotide binding and oligomerization domain (NOD), and a C-terminal leucine-rich repeat (LRR) domain involved in ligand binding [1]. Following ligation of MDP, NOD2 undergoes self-oligomerization and recruitment of receptor interacting serine/threonine kinase RIP2, which, in turn, leads to the activation of nuclear factor-kB (NF-κB) and mitogen-activated protein kinase (MAPK) pathways. The resulting inflammatory response is characterized by the production of proinflammatory cytokines and the activation of antigen-presenting cells [6,7]. While the NOD2-mediated immune response is critical for mounting a successful defense against bacteria, conversely, dysregulation of this response can have deleterious effects. Several genetic variants of NOD2, characterized by aberrant overaction, have been linked to the development of inflammatory disorders, such as Blau syndrome, and cancer [8].

Given the central role of NOD2 in immunosurveillance, as well as its genetic association with inflammatory diseases, modulation of NOD2 with small-molecules has significant clinical potential. Due to their capacity to stimulate innate immunity, as well as contribute to the generation of adaptive immune responses, NOD2 agonists have been widely highlighted as vaccine adjuvants [9]. Notably, MDP was first identified as the active component of Freund’s complete adjuvant [10]. In recent decades, considerable efforts have been made to explore and optimize the structure–activity relationship of MDP derivatives. While only slight modifications of the dipeptide moiety of MDP are permissible, the *N*-acetylmuramic acid carbohydrate moiety offers more opportunities for chemical modifications [11]. For example, it has been shown that the entire carbohydrate ring is dispensable and can be replaced by suitable aromatic/heteroaromatic groups. The resulting compounds, also known as desmuramylpeptides, have shown potent NOD2 agonist activity in vitro and enhanced adjuvant activity in vivo (Figure 1, compound SG8) [12,13,14]. Conversely, the clinical potential of selective NOD2 antagonists is less explored and no compound has yet been introduced into the clinic. Nevertheless, inhibition of NOD2 signaling offers a viable alternative to potent and broad-range anti-inflammatory drugs for treatment of NOD2-associated diseases [8]. For example, the NOD2-mediated innate immune response was highlighted as an important factor in the pathophysiology of atherosclerosis [15,16]. It has also been shown that the proinflammatory response of NOD2 in the liver promotes hepatocarcinogenesis following activation by gut-derived bacterial PAMPs [17]. Although no clinical trials have been conducted to date, combination therapy with NOD2 antagonists and paclitaxel has shown a beneficial anticancer effect in vivo [18,19].

Although a variety of NOD2 agonists and antagonists have been reported, our understanding of the binding modes of these compounds remains limited. In 2016, a crystal structure of rabbit NOD2 in its ADP-bound inactive form was resolved, providing valuable structural insights into the function of NOD2 for the first time [20]. However, to date, no crystal structure of NOD2 in complex with a ligand has been reported, severely hindering rational structure-based drug design approaches. Therefore, the identification of new NOD2 modulators has been limited to ligand-based design approaches and high-throughput screening (HTS) campaigns. Consequently, besides MDP and its structurally closely related derivatives, no other NOD2 agonistic scaffolds have been identified so far [11]. Given that only slight changes to the dipeptide moiety of MDP are permitted, all NOD2 agonists share the predominantly peptide structure of MDP, which is prone to metabolic instability and rapid elimination [21,22]. Similarly, while dual NOD1/2 antagonists, such as benzodiazepine [18], benzofused five-membered sultam [23], quinazoline [24], and indole [25] derivatives have been reported, the only NOD2 selective antagonists discovered to date are based on the benzimidazole structure of GSK669 (Figure 1, compound GSK669 and its derivative SG84 [26]), which was first discovered by an HTS campaign [27]. Therefore, novel scaffolds capable of modulating the activity of NOD2 are required.

In this work, we implemented homology modeling, molecular docking, and pharmacophore modeling to identify novel structural classes of NOD2 modulators. Prioritization of twelve compounds from virtual screening for biological evaluation was based on docking and rescoring using molecular mechanics with a generalized Born and surface area solvation (MM-GBSA) method. Surprisingly, while all hits were devoid of NOD2 agonist activity, two compounds showed an inhibitory effect on the MDP- and SG8-induced activation of NOD2, which was not due to cytotoxicity. Albeit our in silico investigation was initially directed toward the discovery of new NOD2 agonists, the successful discovery of two NOD2-active screening hits demonstrates that our approach is capable of identifying novel NOD2 modulators. The identified scaffolds represent promising starting points for further optimization and discovery of novel NOD2 antagonists.

## 2. Materials and Methods

### 2.1. Virtual Compound Library Preparation

Three virtual compound libraries were prepared for virtual screening: (1) a selected set of experimentally determined NOD2 active compounds, (2) a calculated set of decoy molecules, and (3) a set of commercially available compounds for hit identification via virtual screening with optimized pharmacophore models. For the NOD2 actives library, twelve NOD2 agonists of the muropeptide and desmuramylpeptide structural classes were manually selected from scientific publications (Supplementary Table S1) [3,4,12,13,14,28,29]. A library of 600 decoy molecules was generated based on the structures of these NOD2 agonists by submitting their structures to the DUD-E decoy generator [30]. This resulted in 50 decoy molecules per compound with similar 1D physicochemical properties, but dissimilar 2D topologies in comparison to the 12 active compounds.

A third library of 556,000 compounds from commercial providers was prepared based on the diversity sets from Enamine, Asinex, ChemBridge, Maybridge, LifeChemicals, Vitas-M and KeyOrganics. Libraries were downloaded in SDF format, merged and duplicates removed using the LigandScout Database Merger and Duplicates Remover nodes, as implemented in the Inte:Ligand Expert KNIME Extensions [31].

For each of the three libraries, a maximum of 200 conformations were generated for each molecule using LigandScout’s iCon algorithm with the default “BEST” settings (max. number of conformers per molecules: 200, timeout (s): 600, RMS threshold: 0.8, energy window: 20.0, max. pool size: 4000, max. fragment build time: 30) [32]. Each library was saved in LDB (LigandScout database format) using LigandScout’s idbgen algorithm with default settings (write all properties and remove duplicates).

### 2.2. Pharmacophore Modeling

Ligand-based pharmacophore modeling: Twelve NOD2 agonists of the muropeptide and desmuramylpeptide structural classes (Supplementary Table S1) were used for the creation of five ligand-based pharmacophore models in LigandScout 4.4 Expert [33]. A maximum of 200 conformations were generated for each compound, as described above. The models were generated using the following ligand-based pharmacophore creation settings: Scoring function: pharmacophore fit and atom overlap; Pharmacophore type: shared feature pharmacophore; Feature tolerance scale factor: 1.0; Maximum number of result pharmacophores: 5. A coat of exclusion volume spheres was also generated around the alignment of the ligands. All ligands of the training set were automatically aligned to the generated pharmacophore models. The resulting ligand-based pharmacophore models were inspected visually and tested for their performance in distinguishing the active and decoy molecules.

Structure-based pharmacophore modeling: The best scoring poses of MDP and SG8 were loaded into the structure-based panel of LigandScout 4.4 Expert. For both poses, a structure-based pharmacophore model was generated, which represented the interactions of the ligand with the residues in the binding pocket. The created models were aligned, and a shared feature pharmacophore model was generated, which incorporated only pharmacophore features present in both models. A coat of exclusion spheres was added to prevent steric clashes with the residues in the binding pocket. The hydrophobic feature was marked as optional to reduce the restrictiveness of the model. Finally, the model was tested for performance in distinguishing between active and decoy molecules.

### 2.3. Protein Preparation and Homology Modeling

The homology model of human NOD2 was constructed with the Prime module of the Schrödinger software suite [34]. The 2.34 Å resolution crystal structure of rabbit NOD2 in its ADP-bound inactive state (PDB ID: 5IRN), which has 86% sequence homology to human NOD2, was used as the template [20]. Prior to homology modeling, the template structure was prepared using the Protein Preparation Workflow system [35]. Briefly, force-field atom types and bond orders were assigned, missing side chains were added using Prime, correct protonation states were assigned using a pH value of 7.4, the hydrogen bonding network was optimized to address any overlapping hydrogens, and a restrained minimization with a root mean square deviation (RMSD) value of 0.30 Å using the Optimized Potentials for Liquid Simulations 4 (OPLS4) force-field was performed to relieve any strain and alleviate backbone clashes. Water molecules and ADP were removed from the resulting structure.

Sequence alignment with the human NOD2 target sequence (UniProtKB ID: Q9HC29) was performed in the Multiple Sequence Viewer using the MUSCLE (MUltiple Sequence Comparison by Log- Expectation) algorithm as implemented in Schrödinger software. The construction of the model was performed using the knowledge-based method. As none of the loop regions absent from the template structure were located in the vicinity of the putative ligand-binding site, missing loops were not built. The gaps were capped with *N*-methyl-amide (NMA) groups at the C-termini and acetyl (ACE) groups at the N-termini. The overall quality of the resulting model was evaluated with PROCHECK [36], ERRAT [37] using the Structural Analysis and Verification Server (SAVES, https://saves.mbi.ucla.edu/; Accessed date: 24 November 2021). A Ramachandran plot was generated in Maestro.

### 2.4. Molecular Docking

Three-dimensional models of compounds were built using the LigPrep module of Schrödinger software [38]. Input chiralities were retained, protonation states were generated with Epik at a physiological pH 7.4 [39], and the resulting structures were optimized using the OPLS4 force-field.

A docking receptor grid was generated centering the docking box at the centroid of the following residues: Arg877, Trp931, and Ser933. The size of the docking box was set to 30 × 30 × 30 Å. The hydroxyl group of Ser933 and the thiol group of Cys961 were allowed to rotate during docking.

Compounds were docked with the standard precision (SP) Glide docking methodology [40]. Ligands were docked flexibly, while the protein was kept rigid. Sampling of the ligand conformational space was enhanced by four times, 50,000 poses per ligand were retained after the rough scoring stage and 1000 poses per ligand were kept for energy minimization. Following final docking, 100 poses per ligand were passed to post-docking minimization, and the top ten scoring poses were inspected manually.

### 2.5. Virtual Screening

Selected ligand- and structure-based pharmacophore models were used as queries for virtual screening of a library of 556,000 commercially available compounds. The settings included: Scoring function: relative pharmacophore-fit; Screening mode: match all query features; Retrieval mode: get best matching conformation; Max. number of omitted features: 0; and Check exclusion volumes: checked. The hits were ranked according to their relative pharmacophore fit score. Higher ranking hits have a fit score closer to 1.0. The retrieved hits were transferred to Schrödinger software, prepared using LigPrep and docked as described in the Molecular Docking section above.

### 2.6. Binding Free Energy Calculation

The binding free energies of the top scoring docked poses of all screening hits were calculated with the MM-GBSA method in Prime. For the analysis, the variable-dielectric generalized Born model (VSGB) was used as the continuum solvation model and the OPLS4 was used as the force-field. The docked ligand and all residues within 5.0 Å of the ligand were minimized. The binding free energy was calculated as the difference between the energy of the minimized receptor–ligand complex and the energies of the minimized structures of the unbound ligand and receptor:

Δ G_bind_ = G_complex_ − (G_protein_ + G_ligand_)

### 2.7. Analytical Procedures

The chemical identity of purchased compounds was confirmed by ^1^H-NMR and ^1^H-^1^H COSY 2D NMR on an Avance III spectrometer (Bruker Corporation, Billerica, MA, USA) in DMSO-*d*_6_ with tetramethylsilane as the internal standard. Analytical UHPLC analyses were performed on a Dionex UltiMate 3000 Rapid Separation Binary System (Thermo Fisher Scientific, Waltham, MA, USA). The column used was a Waters Acquity UPLC BEH C18 (1.7 µm, 2.1 × 50 mm), with a flow rate of 0.3 mL/min. The eluent was a mixture of 0.1% trifluoroacetic acid (TFA) in water (A) and acetonitrile (B), with a gradient of (%B): 0–10 min, 5–95%; 10–12 min, 95%; 12–12.5 min, 95–5%. The columns were thermostated at 40 °C. The purity of all biologically tested compounds was >95%.

### 2.8. Cell Culture

HEK-Blue NOD2 cells (Invivogen, San Diego, CA, USA) were cultured according to the manufacturer instructions in Dulbecco’s modified Eagle’s medium (Sigma-Aldrich, St. Louis, MO, USA) supplemented with 10% heat-inactivated fetal bovine serum (Gibco), 2 mM L-glutamine (Sigma-Aldrich), 100 U/mL penicillin (Sigma-Aldrich), 100 µg/mL streptomycin (Sigma-Aldrich), and 100 µg/mL Normocin (Invivogen) for two passages. All subsequent passages were cultured in medium additionally supplemented with 100 µg/mL Zeocin and 30 µg/mL Blasticidin (Invivogen). The cells were incubated in a humidified atmosphere at 37 °C and 5% CO_2_.

### 2.9. Cytotoxicity

HEK-Blue NOD2 cells were seeded (40,000 cells/well) in 96-well plates in 100 µL culture medium and treated with the screening hits (500 µM) or with the corresponding vehicle (0.2% DMSO; control cells). After 18 h of incubation (37 °C, 5% CO_2_), the metabolic activity was assessed using the CellTiter 96 Aqueous One Solution cell proliferation assay (Promega, Madison, WI, USA), according to the manufacturer’s instructions. The experiments were run in duplicates and repeated as two independent biological replicates.

### 2.10. NOD2-NF-κB Reporter Assay

HEK-Blue NOD2 cells were seeded (25,000 cells/well) in 96-well plates in 100 µL HEK-Blue detection medium (Invivogen, San Diego, CA, USA). To test for NOD2 agonism, the cells were treated with the screening hits or with the corresponding vehicle (0.2% DMSO). MDP and SG8 (1 µM) were used as the positive controls. After 18 h of incubation (37 °C, 5% CO_2_), secreted embryonic alkaline phosphatase (SEAP) activity was determined spectrophotometrically as absorbance at 630 nm (BioTek Synergy microplate reader; Winooski, VT, USA). The experiments were run in duplicates and repeated as two independent biological replicates.

To test for NOD2 antagonism, the cells were first pre-treated for 1 h with the screening hits, before the addition of MDP or SG8 (1 µM). After 18 h of incubation (37 °C, 5% CO_2_), SEAP activity was determined as above. The experiments were run in duplicates and repeated as four independent biological replicates.

### 2.11. Statistics

Data analysis was performed using Prism software (version 9; GraphPad Software, CA, USA).

## 3. Results and Discussion

### 3.1. Ligand-Based Pharmacophore Modeling

In our first approach to in silico discovery of novel NOD2 modulators, we employed ligand-based pharmacophore modeling to identify the structural features required for molecular recognition of ligands by NOD2. Twelve representative, highly active NOD2 agonists from the muropeptide and desmuramylpeptide structural classes were selected as a training set (Supplementary Table S1) for the creation of a ligand-based pharmacophore model using LigandScout 4.4 Expert software [33]. Cellular stability studies have shown that the ethyl ester groups of the desmuramylpeptides **S5–S12** primarily allow for cellular internalization and do not actively contribute to NOD2 binding [13]. Therefore, the hydrolyzed free acid forms of these compounds were used to create the model. Up to 200 low-energy conformations were generated for each ligand. Based on their alignment, a model was generated that included only pharmacophore features that were present in the entire training set. Finally, a coat of exclusion spheres was added around the alignment to represent restricted space inaccessible to any potential screening hit.

**Figure 2:**
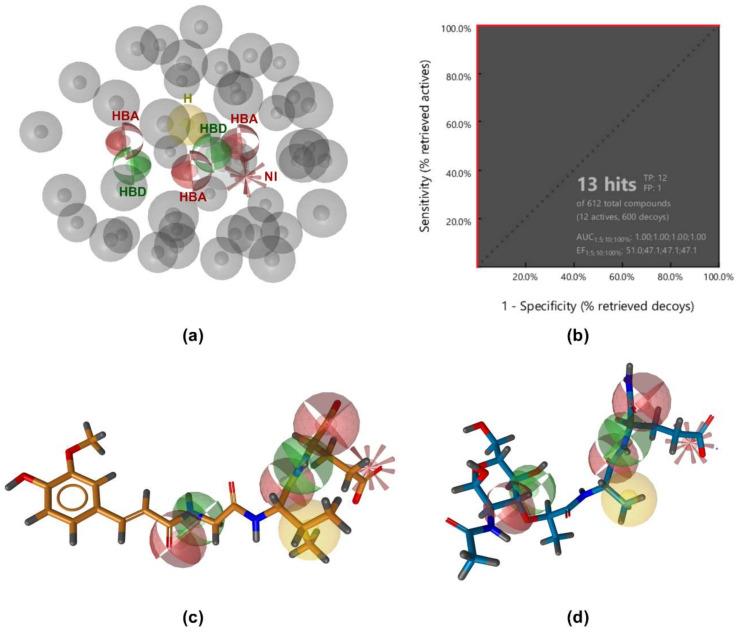
(**a**) Ligand-based pharmacophore model. (**b**) Receiver operating characteristic (ROC) curve from virtually screening 612 compounds (12 actives and 600 generated decoys). TP = true positives; FP = false positives; AUC = area under the curve; EF = enrichment factor. (**c**,**d**) Alignment of SG8 (**c**) and MDP (**d**) with the ligand-based pharmacophore model. The features are as follows: H-bond acceptor (HBA, red sphere), H-bond donor (HBD, green sphere), hydrophobic feature (H, yellow sphere), negative ionizable feature (NI, red star), and exclusion volumes (gray spheres). For clarity, exclusion spheres are not shown in panels (**c**,**d**).

The constructed model, which consisted of seven pharmacophore features (three hydrogen bond acceptors, two hydrogen bond donors, one negative ionizable feature, and one hydrophobic feature; Figure 2a), was then tested for its ability to discriminate between the 12 active and 600 decoy molecules generated by the DUD-E server [30]. The model performed well, retrieving all active compounds and only one decoy (Figure 2b). However, it proved too restrictive and returned no hits when used as a query for virtual screening of a library of 556,000 commercially available compounds. Simplification of this model by omitting some of the pharmacophore features or excluding the exclusion volumes coat improved the number of hits identified, but also reduced the specificity of the model, resulting in a higher number of identified decoys as false positives. Furthermore, there are currently no data on the exact binding modes of NOD2 ligands that would allow for absolute discrimination between essential and redundant pharmacophore features. In the absence of previous data to guide the simplification of the model, key pharmacophore features could therefore be excluded, resulting in a lower sensitivity of the model.

### 3.2. Structure-Based Pharmacophore Modeling

To address the limitations described above and to investigate the potential binding modes of selected NOD2 agonists, a homology model of human NOD2 was first constructed using the crystal structure of rabbit NOD2 (PDB ID: 5IRN) as a template [20]. Although this structure was resolved in its ADP-bound apo form, a potential ligand-binding pocket was identified via mutational studies on the concave surface of the leucine-rich repeat (LRR) domain. Interestingly, an unknown electron density that could not be assigned to any of the molecules used in the purification and crystallization of the protein was found to occupy this pocket. These findings are further supported by surface plasmon resonance (SPR) binding experiments using immobilized MDP and a recombinant functional LRR domain of NOD2 [41]. In both studies, Arg877, Trp931, and Ser933 were highlighted as critical residues for MDP recognition.

Analysis of the aligned sequences of rabbit and human NOD2 revealed a high 86% sequence homology and complete conservation of the putative ligand-binding residues. A homology model was built using the Prime module of the Schrödinger software suite (Supplementary Figure S1) and the quality of the model was validated using PROCHECK and ERRAT. Assessment of the generated Ramachandran plot shows that only four residues were in the disallowed region and none of them were in the vicinity of the binding pocket (Supplementary Figure S2) making our model reliable for structure-based modeling.

**Figure 3:**
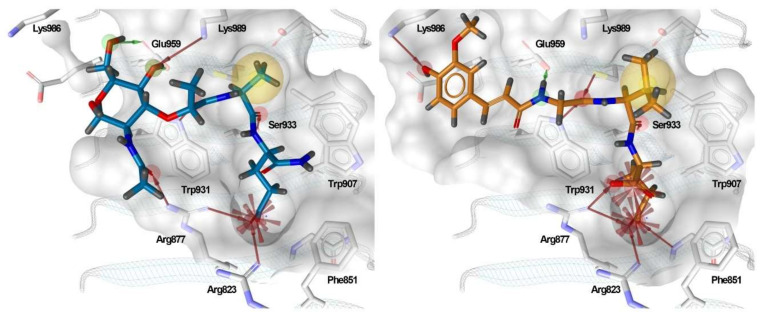
Predicted binding modes of MDP (blue sticks) and SG8 (orange sticks). Overlaid are the generated structure-based pharmacophore models based on the interactions between the ligand and the residues in the binding site. The features are as follows: H-bond acceptor (green arrow), H-bond donor (red arrow), hydrophobic feature (yellow sphere), and negative ionizable feature (red star).

A docking grid was generated, centering the box on Arg877, Trp931, and Ser933, and MDP and SG8 were docked as representative potent NOD2 agonists of the muropeptide and desmuramylpeptide structural classes. The predicted binding models showed a striking similarity between the orientations of the dipeptide moieties of both molecules (Figure 3). The negatively charged carboxylate groups of D-glutamic acid and D-isoglutamine anchored the compounds in the pocket via electrostatic interactions with Arg823 and Arg877. The carbonyl oxygens of L-alanine and L-valine in MDP and SG8, respectively, formed a hydrogen bond with Ser933, while the hydrophobic side-chains of these amino acids fit tightly into the hydrophobic side pocket formed by Trp887, Val915, Cys941, and other adjacent residues. Additional hydrogen bonding of both compounds was also observed with Trp931 and Glu959. In addition, both compounds also exhibited unique interactions. The *N*-acetyl group of MDP formed an additional hydrogen bond with Arg877, which is consistent with previous docking and SPR studies that indicated that Arg877 interacts with both the dipeptide and the carbohydrate moieties of MDP [41]. Conversely, the *trans*-ferulic acid moiety of SG8 extends beyond the main pocket and forms an additional hydrogen bond with Lys986, while the methoxy group is oriented towards a hydrophobic side pocket. These data agree with our earlier in vitro investigations of desmuramylpeptide NOD2 activity, where a derivative with an unsubstituted aromatic ring exhibited weaker but still nanomolar activity. This suggests that the interactions of the hydroxy and methoxy groups are not essential for NOD2 activation but do contribute to tighter binding [13].

**Figure 4:**
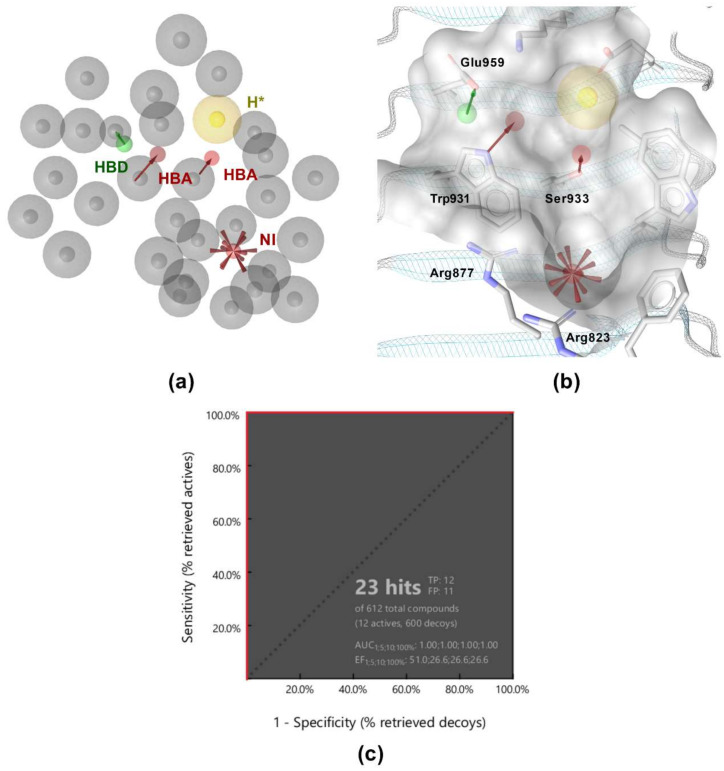
(**a**) Shared features structure-based pharmacophore model. The features are as follows: H-bond acceptor (HBA, red arrow), H-bond donor (HBD, green arrow), hydrophobic feature (H, yellow sphere), negative ionizable feature (NI, red star), and exclusion volumes (gray spheres). *Hydrophobic feature is marked as optional. (**b**) Overlay of the structure-based pharmacophore model with the NOD2 ligand-binding pocket. (**c**) Receiver operating characteristic (ROC) curve from virtually screening 612 compounds (12 actives and 600 generated decoys). TP = true positives; FP = false positives; AUC = area under the curve; EF = enrichment factor.

The complementary nature of the interaction patterns of MDP and SG8 prompted us to construct a structure-based pharmacophore model that would accurately represent the joint interaction fingerprint of both ligands. In contrast to the ligand-based approach described above, structure-based pharmacophore modeling is based on the probing of possible interaction points between the ligand and the protein. A pharmacophore feature is only added to the model when a complementary binding partner in the correct geometry is identified in the binding site. A structure-based pharmacophore model was built for the docked poses of MDP and SG8 in LigandScout. Following their alignment, only features present in both models were retained and a coat of exclusion spheres, based on the positions of the binding site residues, was added to prevent steric clashes of potential screening hits with the protein. The constructed model incorporated five pharmacophore features: a negative ionizable feature in the vicinity of the positively charged Arg823 and Arg877, two hydrogen bond acceptors predicted to interact with Trp931 and Ser933, a hydrogen bond donor predicted to interact with Glu959, and a hydrophobic feature, which was designated as optional to reduce the restrictiveness of the model (Figure 4a,b). Namely, it has been reported previously that replacement of L-alanine in MDP with its more hydrophilic analogs, such as L-serine, reduces but does not abolish the activity of NOD2, suggesting that this feature is dispensable for NOD2 activation [28].

The validation library of 12 active and 600 decoy molecules was used again to evaluate the performance of the model. As illustrated by Figure 4c, the model correctly identified all active compounds, while it also retrieved eleven decoys as false positives. Despite the slightly lower specificity in comparison to the ligand-based pharmacophore model, the enrichment factor (EF = 51) was still satisfactory. Importantly, the model was less restrictive and had a higher hit rate when used as a query for virtual screening of the library of commercially available compounds.

### 3.3. Virtual Screening

The optimized structure-based pharmacophore model found 79 hits from the library of 556,000 compounds, corresponding to an overall hit rate of 0.014%. In an additional filtering step, the hits were docked to the homology model using the same parameters as for the docking of MDP and SG8. Because the scoring functions of docking algorithms sacrifice accuracy in favor of computational efficiency, the calculated docking scores only allow for rough discrimination between active and inactive compounds and generally correlate poorly with experimental results. To reduce the proportion of false positive hits, the docked poses were therefore rescored with the MM-GBSA method, which estimates binding free energies using molecular mechanics and continuum solvent models [42].

Twelve compounds were prioritized for purchase and experimental evaluation based on the calculated binding affinities, scaffold diversity, and manual inspection of the predicted binding modes. Only compounds predicted to interact with at least three of the four residues, identified by our structure-based pharmacophore model, were considered for evaluation. Table 1 summarizes the structures of the purchased hits with their pharmacophore fit scores, docking scores, and binding free energies calculated using the MM-GBSA method. All compounds contained a carboxylic acid group to match the negative ionizable feature of the pharmacophore model. Only **11** and **12** matched the optional hydrophobic feature, as indicated by the higher relative pharmacophore fit scores (0.916–0.929).

### 3.4. In Vitro Biological Evaluation of Virtual Screening Hits

**Figure 5:**
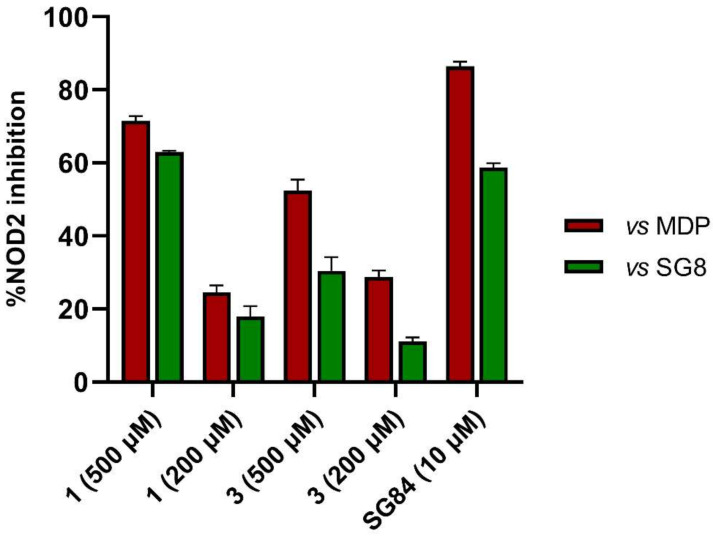
The NOD2 antagonistic activity of virtual screening hits **1** and **3**. HEK-Blue NOD2 cells were pre-treated for 1 h with **1** or **3**, before 18 h stimulation with SG8 (1 μM) or MDP (1 μM). A previously reported NOD2 antagonist SG84 was used as the positive control [26]. The data are means ± SEM of four independent experiments and are shown as % inhibition in comparison to the response observed after stimulation with SG8 or MDP alone.

The purchased compounds were evaluated using the commercially available HEK-Blue NOD2 reporter cell line. This cell line, based on HEK293 cells, stably expresses human NOD2 as well as an NF-κB-inducible secreted embryonic alkaline phosphatase (SEAP) reporter gene. Activation of NOD2, and subsequently of NF-κB, induces the expression and secretion of SEAP, the levels of which can be detected colorimetrically. Interestingly, although none of the compounds activated NOD2 at the highest concentration tested (500 μM; Supplementary Figure S3), two compounds, **1** and **3**, exhibited a weak inhibitory effect on the activation of NOD2 by MDP and SG8 (Figure 5). Pre-treatment of HEK-Blue NOD2 cells with **1** (500 μM) reduced the observed NF-κB transcriptional activity after stimulation with MDP and SG8 by 71% and 63%, respectively. This NOD2 antagonistic activity was comparable to a 10 μM concentration of a previously reported benzimidazole NOD2 antagonist SG84 [26]. Slightly weaker antagonism of NOD2 was observed by pre-treatment with **3** (500 μM), which reduced the responses elicited by MDP and SG8 by 52% and 30%, respectively. Both compounds displayed dose-dependent antagonism of NOD2, with some activity still evident at 200 μM. Importantly, the observed antagonistic effect was not due to cytotoxicity. Neither compound reduced the measured metabolic activity of HEK-Blue NOD2 cells at the highest concentration tested (500 μM), as determined by the (3-(4,5- dimethylthiazol-2-yl)-5-(3-carboxymethoxyphenyl)-2-(4-sulfo-phenyl)-2*H*-tetrazolium) (MTS) method (Supplementary Figure S4).

### 3.5. Binding Modes of Compounds **1** and **3** in the Putative NOD2 Ligand-Binding Pocket

Our docking study identified four key pharmacophoric features shared by MDP and SG8. Namely, electrostatic interactions within the positively charged pocket formed by Arg823 and Arg877, and three hydrogen bonds with Trp931, Ser933, and Glu959. The docked poses of **1** and **3** were examined in detail and compared with those of MDP and SG8 to investigate whether the predicted binding modes were consistent with the constructed pharmacophore model. No constraints were defined during the docking protocol in order not to influence the orientation of the docked ligands.

**Figure 6:**
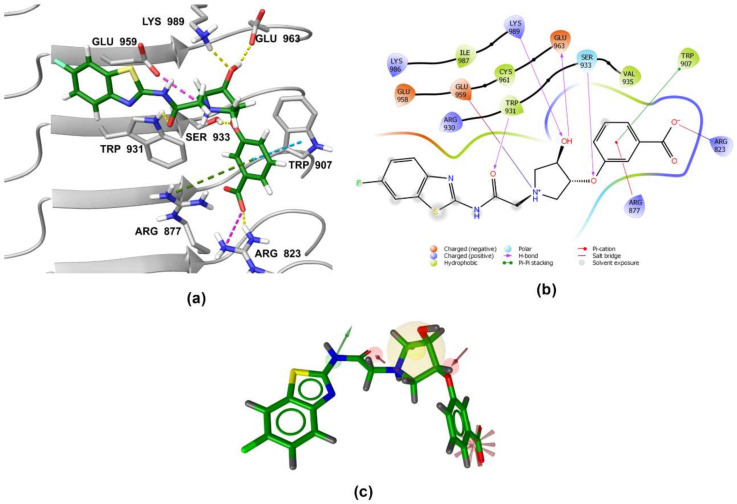
(**a**) Predicted binding mode of **1** in the human NOD2 homology model. For clarity, only amino acid side-chains interacting with the docked ligands are shown as grey sticks. The interactions are colored as follows: hydrogen bond (yellow dashed line), salt bridge (purple dashed line), π-π stacking (blue dashed line), cation-π interaction (green dashed line). (**b**) 2D diagram of interactions between **1** and the binding pocket residues. (**c**) Alignment of the LigandScout-generated conformation of **1** with the structure-based pharmacophore model.

**Figure 7:**
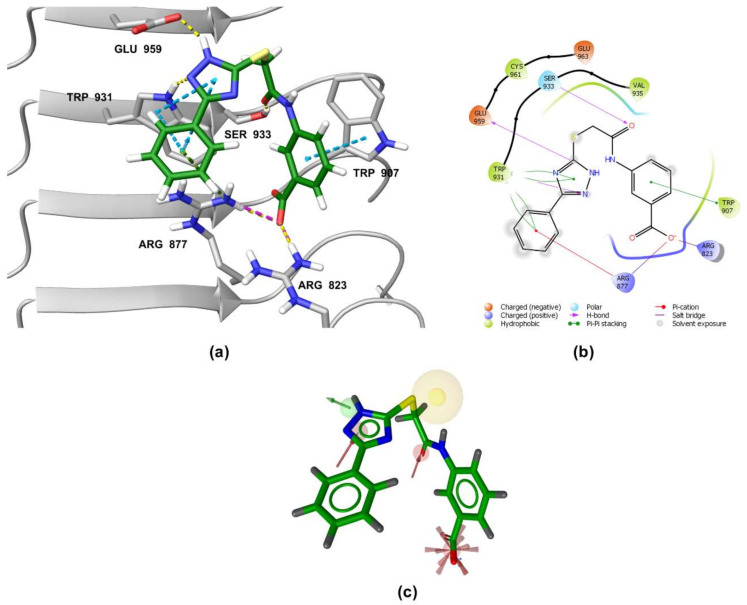
(**a**) Predicted binding mode of **3** in the human NOD2 homology model. For clarity, only amino acid side-chains interacting with the docked ligands are shown as grey sticks. The interactions are colored as follows: hydrogen bond (yellow dashed line), salt bridge (purple dashed line), π-π stacking (blue dashed line), cation-π interaction (green dashed line). (**b**) 2D diagram of interactions between **3** and the binding pocket residues. (**c**) Alignment of the LigandScout-generated conformation of **3** with the structure-based pharmacophore model.

Both screening hits contain an aromatic carboxylate motif that is predicted to occupy the positively charged pocket where it interacts with Arg823 and Arg877 via electrostatic or cation-π interactions and with Trp907 via π-π stacking (Figure 6 and Figure 7) A hydrogen bond is formed between Ser933 and the ether oxygen of **1**, while the amide carbonyl oxygen forms a hydrogen bond with Trp931. In addition, the hydroxyl group on the five-membered ring forms two hydrogen bonds with Glu963 and Lys989. Interestingly, although the predicted binding conformation of **1** correlated well with its orientation in the pharmacophore model (Figure 6c), the nature of the identified interaction with Glu959 differed between the two methods and, consequently, between the docked poses of **1** and MDP/SG8. Namely, instead of hydrogen bonding formed between Glu959 and MDP/SG8, an electrostatic interaction forms between this residue and the cationic tertiary amine of **1**.

A similar binding orientation was predicted for **3** (Figure 7a,b). In addition to the electrostatic, cation-π, and π-π interactions of the aromatic carboxylate motif with Arg823 and Arg877, three hydrogen bonds form, corresponding to the hydrogen bonding network formed by MDP and SG8. Namely, Ser933 interacts with the carbonyl oxygen of **3**, while its heteroaromatic imidazole ring forms two additional hydrogen bonds with Trp931 and Glu959. Remarkably, the conformation of **3** generated by LigandScout, which was identified during virtual screening as matching the pharmacophore model, is nearly identical to the pose of **3** generated during docking (Figure 7c).

**Figure 8:**
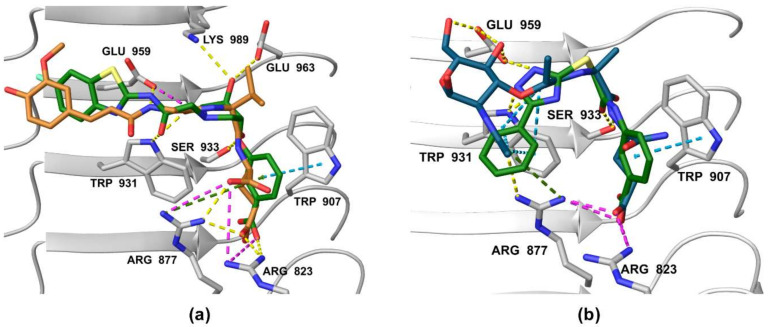
Comparison of docked poses of (**a**) **1** (green) and SG8 (orange), and (**b**) **3** (green) and MDP (blue). The interactions are as follows: hydrogen bond (yellow dashed line), salt bridge (purple dashed line), π-π stacking (blue dashed line), cation-π interaction (green dashed line).

Analysis of the docked poses of **1** and **3,** therefore, showed that both screening hits were able to form a similar interaction fingerprint compared to the compounds that served as the basis for constructing the pharmacophore model. Interestingly, the binding mode of **1** is complementary to that of SG8, while the docked pose of **3** correlates better with that of MDP (Figure 8). All four compounds show highly similar positioning of the pivotal negatively charged carboxylic acid in the positively charged pocket and of the hydrogen bond acceptor interacting with Ser933. However, while both screening hits interact with a similar set of residues within the binding site, there are some differences in the nature of these interactions that may provide an explanation for the contrasting modes of action of these NOD2 modulators. For example, in addition to the electrostatic interaction of **1** with Glu959 mentioned above, both screening hits form a π-π stacking interaction with Trp907, an interaction not available to the D-isoGln and D-Glu moieties of MDP and SG8, respectively. In contrast to the hydrogen bond formed between Arg877 and the *N*-acetyl group of the carbohydrate moiety of MDP, **3** interacts with this residue via a cation-π interaction. Finally, the positioning of the hydrogen bond acceptors interacting with Trp931 is different between the screening hits and MDP/SG8.

It is known that upon binding MDP or its derivatives, including SG8, NOD2 undergoes extensive ligand-induced conformational changes, which ultimately lead to downstream signal transduction. We therefore hypothesize that although our structure-based pharmacophore modeling approach correctly identified the structural features responsible for binding to the putative NOD2 ligand-binding site, it lacks the fine structural details that would enable NOD2 activation. SPR and backscattering interferometry (BDI) experiments have previously demonstrated that the individual components of MDP (dipeptide and carbohydrate) bind to purified NOD2 with nanomolar affinity but lack the NOD2 activating capacity of the entire MDP molecule [43]. This study has thus shown that strong binding is necessary but not sufficient for NOD2 agonism. Similarly, conjugates of paclitaxel with an MDP analogue containing a muramic acid moiety synergistically induced TNF-α and IL-12 production in murine peritoneal macrophages [44]. Interestingly, when the muramic acid moiety was replaced with a cinnamic acid derivative, the resulting conjugate exhibited NOD2 antagonist activity with potent antitumor activity due to suppression of the inflammatory tumor microenvironment [19]. This shows that subtle structural differences can influence the mode of action of NOD2 modulators.

One of the major limitations in the rational design of NOD2 modulators remains the lack of a crystal structure of NOD2 in complex with a ligand. While many potent NOD2 agonists have been developed, the vast majority is closely based on the structure of MDP and other muropeptides. As exemplified by our initial ligand-based pharmacophore modelling approach, their low structural diversity hinders the elucidation of the key structural features involved in NOD2 binding. Without means to discriminate between redundant and essential features, the generated pharmacophore model contained too many features and was, therefore, deemed too restrictive for virtual screening. We successfully circumvented these limitations by employing a hybrid docking-pharmacophore modelling approach. Due to the low hit rate of the constructed structure-based model, it performed well as a primary screen to reduce the number of potentially active compounds and consequently to reduce the computational burden of the subsequent docking and MM-GBSA rescoring steps. Notably, as can be seen in Table 1, the MM-GBSA-predicted binding energies of both active hits were among the three best of all the purchased screening hits, showing that the MM-GBSA rescoring step provided a better correlation with the experimental results compared to the pharmacophore fit and Glide docking scores alone. Although the MM/GBSA method is computationally demanding, its use is, therefore, worthwhile, provided that an appropriate pre-filtering step, such as our pharmacophore model, is applied to the screening library prior to its use.

Further experimental studies would be required to conclusively determine whether **1** and **3** interact directly with NOD2 or exert their inhibitory effect on one of the downstream signaling proteins. However, given their low structural complexity, the two novel scaffolds represent valuable starting points for further optimization to expand the currently limited library of NOD2 antagonists.

## 4. Conclusions

In conclusion, we successfully deploy here a hybrid docking and pharmacophore modelling approach to NOD2 ligand discovery. Docked poses of previously reported NOD2 agonists were used as the basis for the construction of a structure-based pharmacophore model. Two compounds, **1** and **3**, which were identified by the constructed model, exhibited an inhibitory effect on MDP- and SG8-induced NOD2 activation, thus providing valuable novel NOD2 antagonist scaffolds suitable for further optimization. Interestingly, the mode of action of the compounds used in the construction of the model and the identified virtual screening hits do not match. This suggests that, while our approach has identified the structural requirements for NOD2 binding, it does not provide sufficient structural data for NOD2 activation. Nevertheless, it represents a promising starting point for further optimization and, to the best of our knowledge, is the first successful virtual screening campaign for the discovery of NOD2 modulators.

## Figures and Tables

**Table 1:** Prioritized virtual screening hits with relative pharmacophore fit scores, docking scores, and MM-GBSA predicted binding energies.

Cpd.	Structure	Relative Pharmacophore Fit Score ^1^	Docking Score (kcal/mol)	MM-GBSA Binding Energy (kcal/mol)
MDP	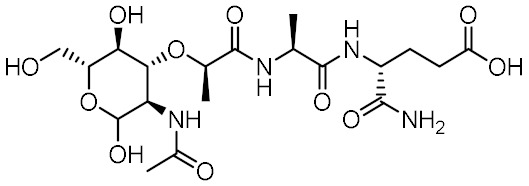	0.955	−6.662	−58.60
SG8	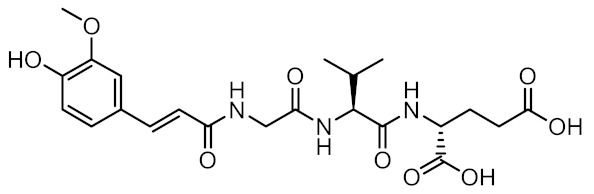	0.976	−6.677	−53.68
**1**	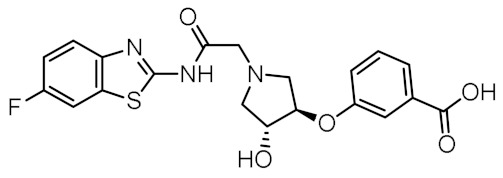	0.736	−5.846	−67.06
**2**	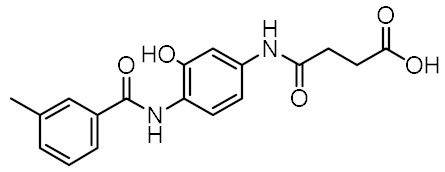	0.764	−6.164	−57.74
**3**	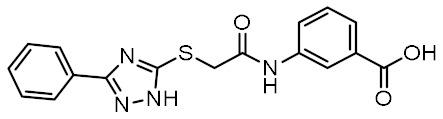	0.759	−5.473	−54.87
**4**	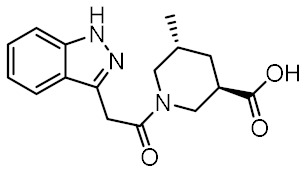	0.756	−6.118	−52.05
**5**	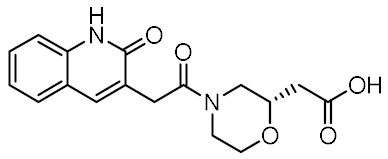	0.759	−7.051	−50.96
**6**	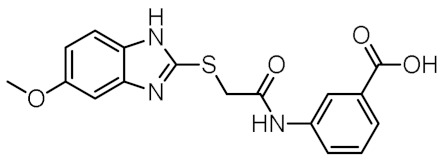	0.785	−5.694	−50.36
**7**	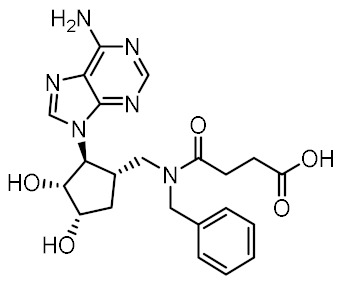	0.778	−5.96	−50.13
**8**	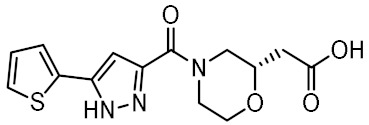	0.747	−5.501	−46.51
**9**	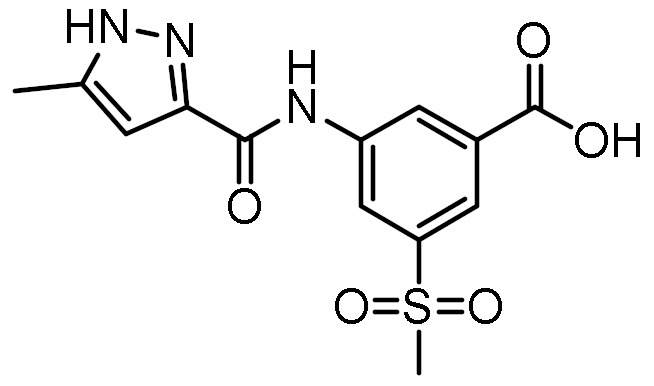	0.747	−6.129	−46.43
**10**	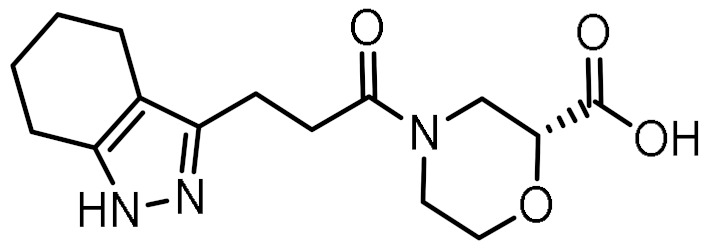	0.742	−5.793	−41.98
**11**	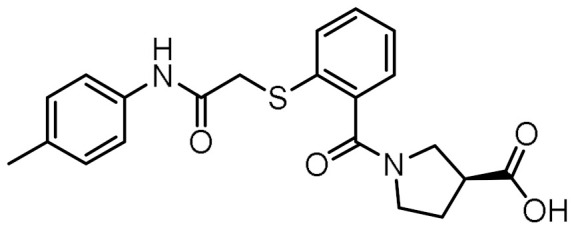	0.916	−5.021	−40.09
**12**	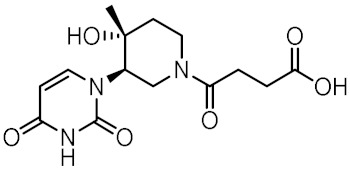	0.929	−5.559	−34.63

^1^ Higher relative pharmacophore fit scores indicate a closer fit to the model.

## Data Availability

Data is contained within the article and Supplementary Materials.

## Supplementary Materials

The following supporting information can be downloaded at: https://www.mdpi.com/article/10.3390/biom12081054/s1, Table S1: Library of NOD2 active compounds. Figure S1: Structure of the homology model of NOD2, Figure S2: Ramachandran plot of the constructed homology model., Figure S3: NOD2 agonism assay, Figure S4: Cytotoxicity assay.
