# Supplementary material for: Novel Scaffolds for Modulation of NOD2 Identified by Pharmacophore-Based Virtual Screening
Source: Biomolecules. 2022 Jul 29;12(8):1054. doi: 10.3390/biom12081054 (PMC9405794; doi:10.3390/biom12081054)
Supplement: Supplementary file 1 [file biomolecules-12-01054-s001.zip › biomolecules-1792846-supplementary.pdf]

biomolecules

MDPI

Supplementary Materials

Novel scaffolds for modulation of NOD2 identified by pharmacophore-based virtual screening

Samo Guzelj [1], Tihomir Tomašič [1] and Žiga Jakopin [1,*]

[1] Faculty of Pharmacy, University of Ljubljana, Aškerčeva cesta 7, 1000 Ljubljana, Slovenia; samo.guzelj@ffa.uni-lj.si (S.G.); tihomir.tomasic@ffa.uni-lj.si (T.T.)
* Correspondence: ziga.jakopin@ffa.uni-lj.si; Tel.: +386 1 4769 646

Table S1. Structures of NOD2 agonists **S1-S12** used as a training set for ligand-based pharmacophore model generation.

Cpd.	Structure	Ref.
**S1** (MDP)	O=C(CC[C@@H](NC([C@@H](NC([C@@H](C)O[C@H]1[C@H](O)[C@@H](CO)O[C@H](O)[C@@H]1NC(C)=O)=O)C)=O)C(N)=O)O	[1–3]
**S2**	O=C(CC[C@@H](NC([C@@H](NC([C@@H](C)O[C@H]1[C@H](O)[C@@H](CO)O[C@H](O)[C@@H]1NC(C)=O)=O)C)=O)C(O)=O)O	[3]
**S3**	O=C(CC[C@@H](NC([C@@H](NC([C@@H](C)O[C@H]1[C@H](O)[C@@H](CO)O[C@H](O)[C@@H]1NC(CO)=O)=O)C)=O)C(N)=O)O	[4]
**S4**	O=C(CC[C@@H](NC([C@@H](NC([C@@H](C)O[C@H]1[C@H](O)[C@@H](CN)O[C@H](O)[C@@H]1NC(C)=O)=O)C)=O)C(N)=O)O	[4]
**S5**	CC(C)[C@@H](C(N[C@@H](C(O)=O)CCC(O)=O)=O)NC(CNC(/C=C/C1=CC=C(C(C)C)C=C1)=O)=O	[5]

**S6**	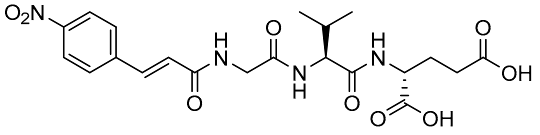<br>CC(C)[C@@H](C(N[C@@H](C(O)=O)CCC(O)=O)=O)NC(CNC(/C=C/C1=CC=C([N+]([O-])=O)C=C1)=O)=O	[5]
**S7**	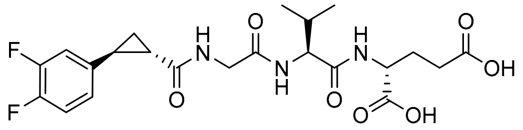<br>CC(C)[C@@H](C(N[C@@H](C(O)=O)CCC(O)=O)=O)NC(CNC([C@@H]1[C@@H](C2=CC(F)=C(F)C=C2)C1)=O)=O	[5]
**S8**	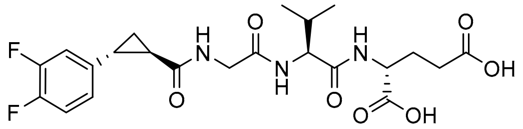<br>CC(C)[C@@H](C(N[C@@H](C(O)=O)CCC(O)=O)=O)NC(CNC([C@H]1[C@H](C2=CC(F)=C(F)C=C2)C1)=O)=O	[5]
**S9 (SG8)**	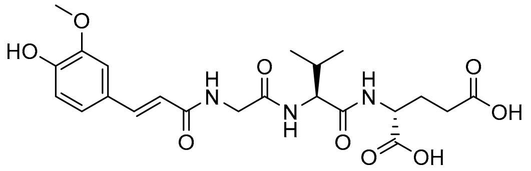<br>CC(C)[C@@H](C(N[C@@H](C(O)=O)CCC(O)=O)=O)NC(CNC(/C=C/C1=CC(OC)=C(O)C=C1)=O)=O	[6]
**S10**	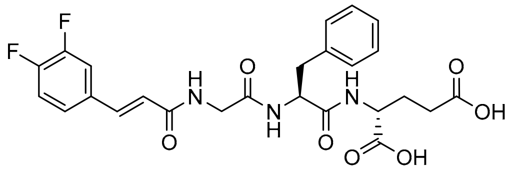<br>O=C(N[C@@H](C(O)=O)CCC(O)=O)[C@H](CC1=CC=CC=C1)NC(CNC(/C=C/C2=CC(F)=C(F)C=C2)=O)=O	[6]
**S11**	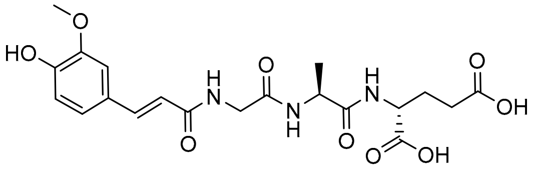<br>C[C@@H](C(N[C@@H](C(O)=O)CCC(O)=O)=O)NC(CNC(/C=C/C1=CC(OC)=C(O)C=C1)=O)=O	[6]
**S12**	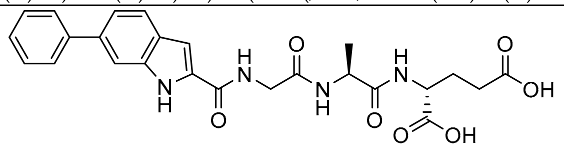<br>C[C@@H](C(N[C@@H](C(O)=O)CCC(O)=O)=O)NC(CNC(C1=CC(C=C2)=C(N1)C=C2C3=CC=CC=C3)=O)=O	[7]

Supplementary Figures

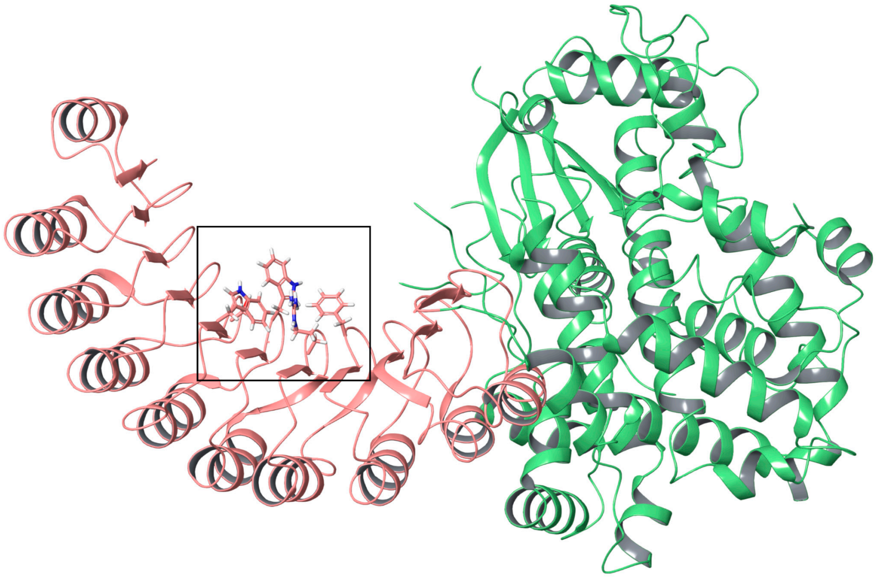

Figure S1. Structure of the homology model of human NOD2, comprised of two domains: the nucleotide-binding oligomerization domain (NOD; 215–764, green) and the leucine-rich repeat domain (LRR; 765–1040). The caspase activation and recruitment domains (CARDs; 1–214) were not modeled. The putative MDP binding site is highlighted by a rectangle.

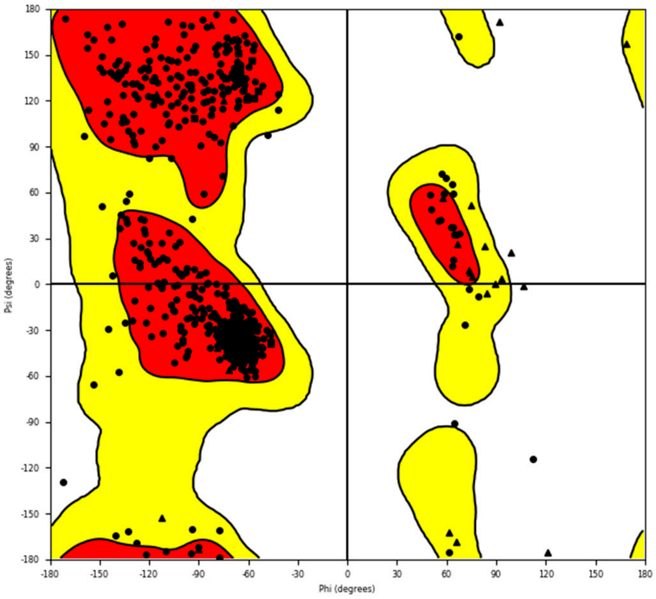

Figure S2. Ramachandran plot of the constructed homology model. Glycine is plotted as triangles, proline is plotted as squares, and all other residues are plotted as circles. The orange regions are the favored regions, the yellow regions are the allowed regions, and the white regions are the disallowed regions. 4 non-glycine residues (0.5% of 746 total residues): Ala525, Ala597, Cys632, and Ile722 are located in the disallowed region.

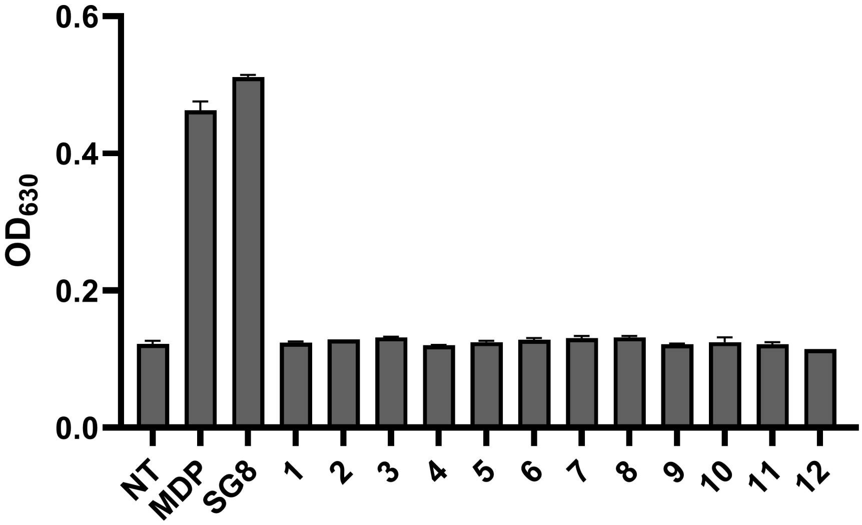

Figure S3.. None of the screening hits exhibited NOD2 agonistic activity. SEAP activity in the HEK-Blue NOD2 cell supernatants was determined after 18 h treatment with the screening hits (500 μM), MDP (1 μM), **SG8** (1 μM), or vehicle (0.1% DMSO; NT). Data are shown as optical densities at 630 nm and are means ± SEM of two independent experiments.

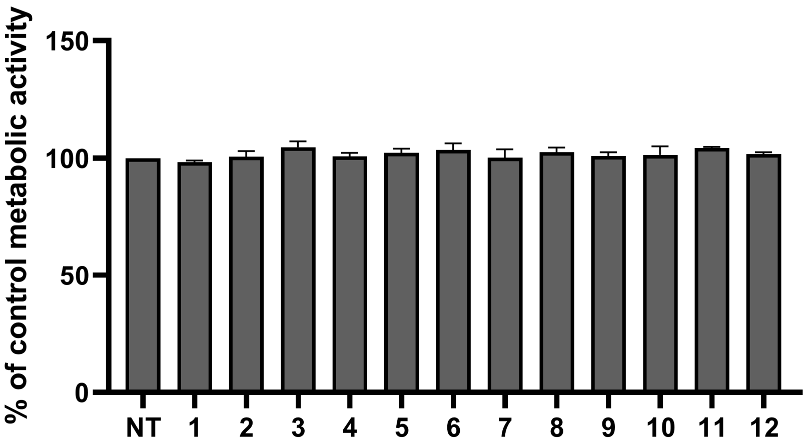

Figure S4. Metabolic activities of HEK-Blue NOD2 cells after 18 h treatment with screening hits (500 μM). The results are shown relative to that of the untreated control (0.1% DMSO; NT). Data are means ± SEM of two independent experiments.

NMR spectra

Compound **1**: ^{1}H, 400 MHz, DMSO-d_6

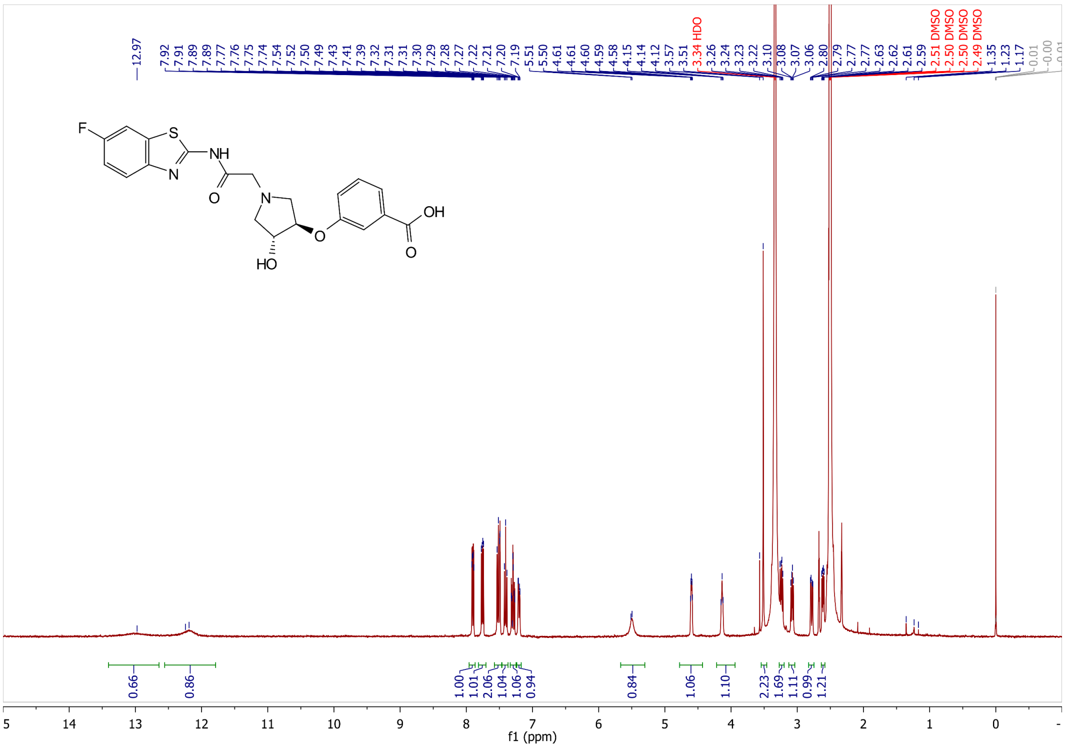

Compound **1**: ^{1}H-^{1}H COSY, 400 MHz, DMSO-d_6

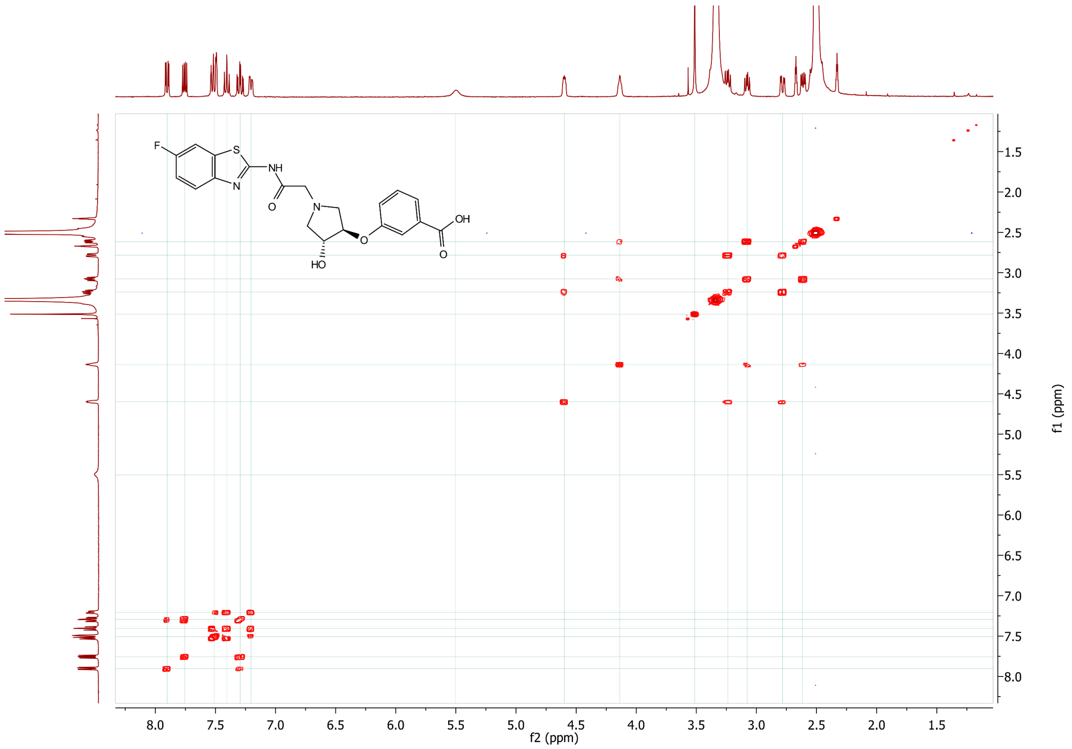

Compound **3**: ^{1}H, 400 MHz, DMSO-d_6

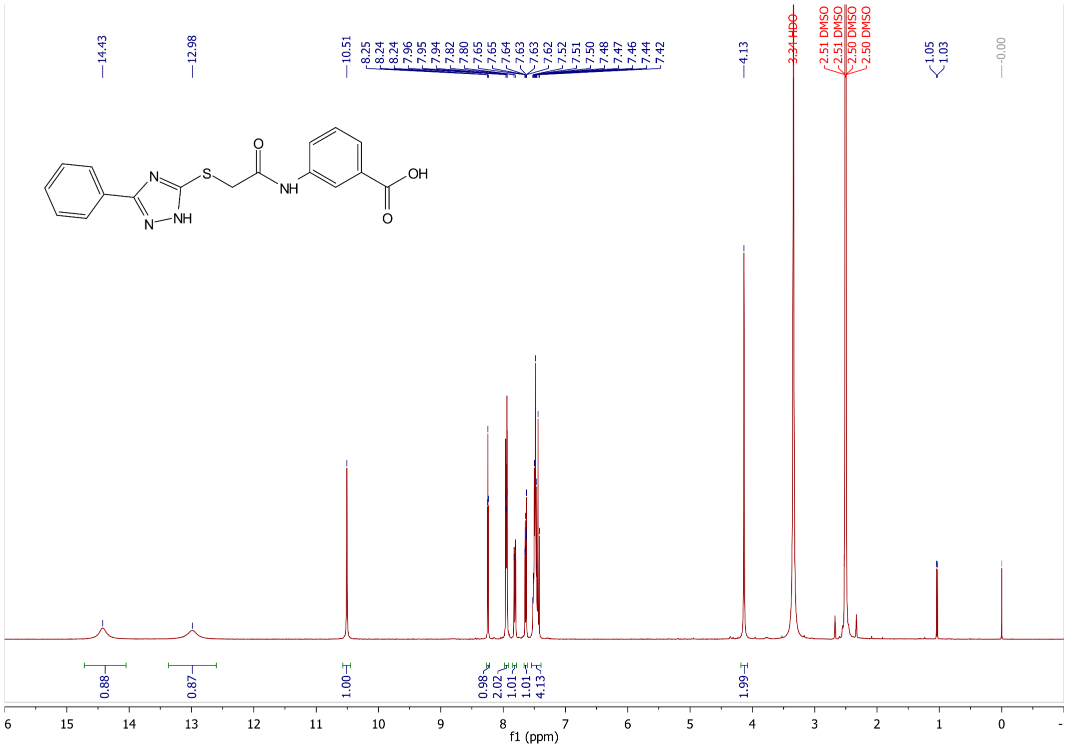

Compound **3**: ^{1}H-^{1}H COSY, 400 MHz, DMSO-d_6

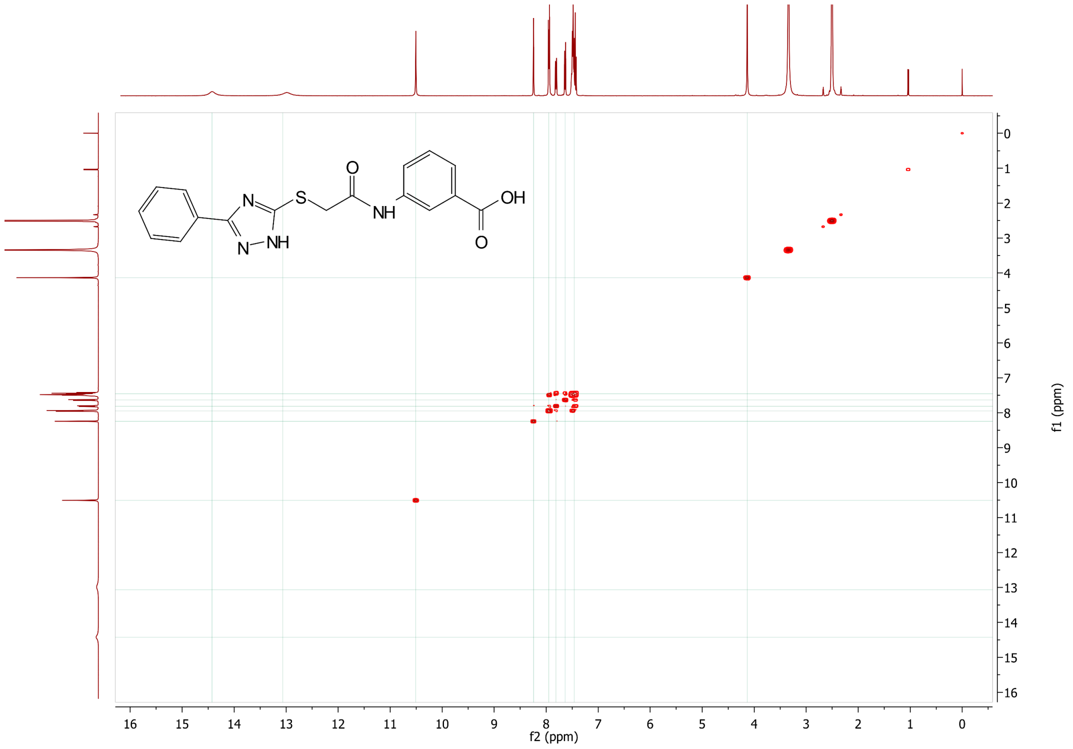

UHPLC traces

Compound **1**

Chromatogram and Results

Injection Details

Injection Name:	**Ax3**	*Run Time (min):*	**12,50**
Processing Method:	**Quantitative**	*Injection Volume:*	**5,00**
Injection Date/Time:	**24.feb..22 15:14**	*Wavelength:*	**254**

Chromatogram

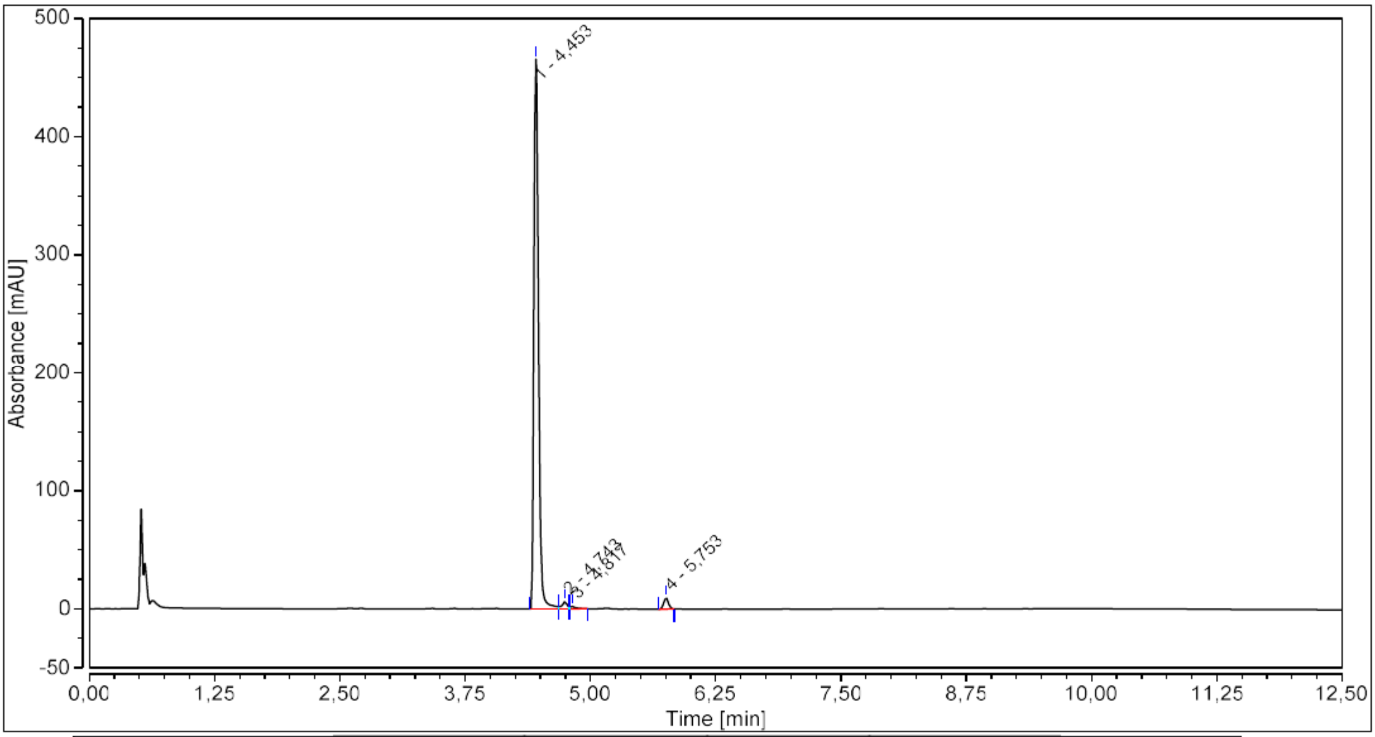

Integration Results

No.	Retention Time min	Area mAU*min	Height mAU	Relative Area %	Relative Height %
1	4,453	24,641	465,970	96,01	96,27
2	4,743	0,357	6,121	1,39	1,26
3	4,817	0,140	2,069	0,55	0,43
4	5,753	0,526	9,871	2,05	2,04
**Total:**		**25,664**	**484,031**	**100,00**	**100,00**

Compound **3**

Chromatogram and Results

Injection Details

Injection Name:	**VM2**	*Run Time (min):*	**12,50**
Processing Method:	**Quantitative**	*Injection Volume:*	**5,00**
Injection Date/Time:	**24.feb..22 15:32**	*Wavelength:*	**254**

Chromatogram

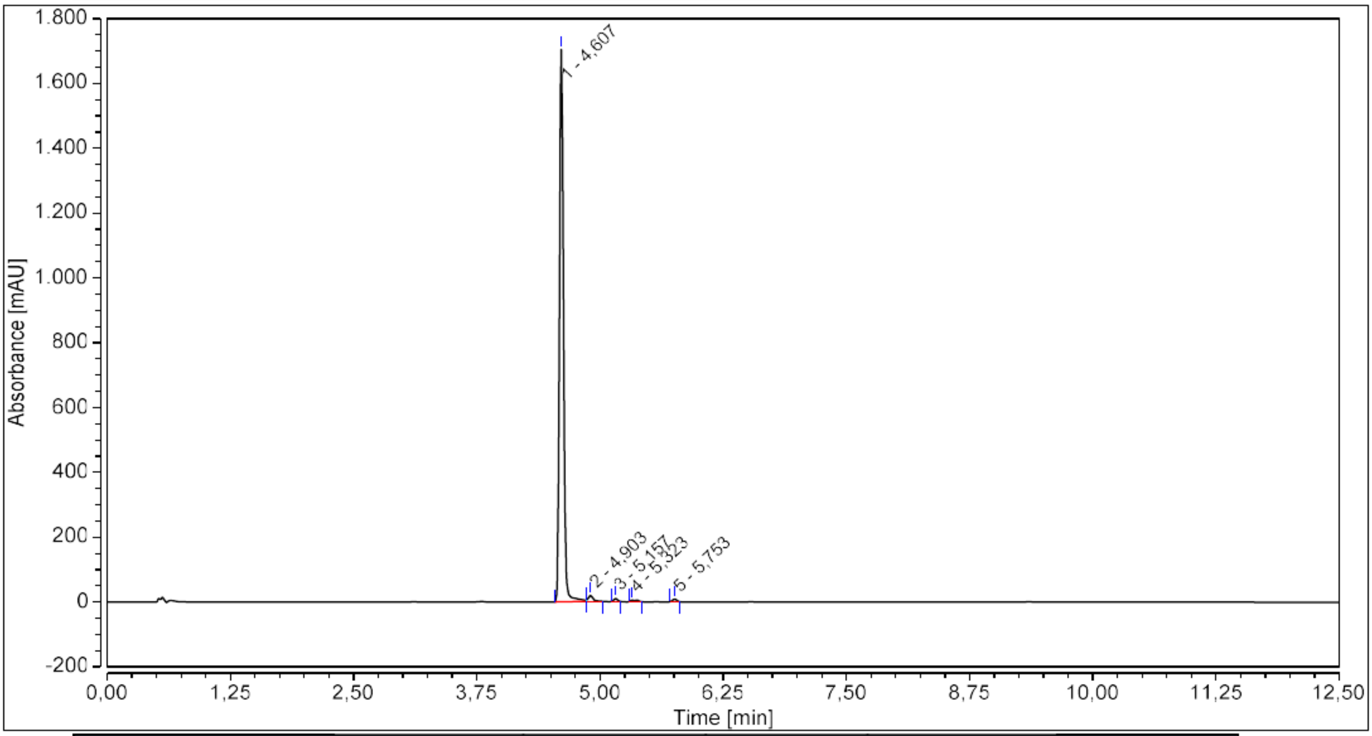

Integration Results

No.	Retention Time min	Area mAU*min	Height mAU	Relative Area %	Relative Height %
1	4,607	78,789	1703,841	97,31	97,60
2	4,903	0,988	18,940	1,22	1,08
3	5,157	0,394	9,601	0,49	0,55
4	5,323	0,413	5,413	0,51	0,31
5	5,753	0,382	7,927	0,47	0,45
**Total:**		**80,966**	**1745,721**	**100,00**	**100,00**

References

1. Girardin, S.E.; Boneca, I.G.; Viala, J.; Chamaillard, M.; Labigne, A.; Thomas, G.; Philpott, D.J.; Sansonetti, P.J. Nod2 Is a General Sensor of Peptidoglycan through Muramyl Dipeptide (MDP) Detection. *J. Biol. Chem.* **2003**, *278*, 8869–8872, doi:10.1074/jbc.C200651200.
2. Inohara, N.; Ogura, Y.; Fontalba, A.; Gutierrez, O.; Pons, F.; Crespo, J.; Fukase, K.; Inamura, S.; Kusumoto, S.; Hashimoto, M.; et al. Host Recognition of Bacterial Muramyl Dipeptide Mediated through NOD2: Implications for Crohn's Disease. *J. Biol. Chem.* **2003**, *278*, 5509–5512, doi:10.1074/jbc.C200673200.
3. Rubino, S.J.; Magalhaes, J.G.; Philpott, D.; Bahr, G.M.; Blanot, D.; Girardin, S.E. Identification of a Synthetic Muramyl Peptide Derivative with Enhanced Nod2 Stimulatory Capacity. *Innate Immun.* **2013**, *19*, 493–503, doi:10.1177/1753425912471691.
4. Melnyk, J.E.; Mohanan, V.; Schaefer, A.K.; Hou, C.W.; Grimes, C.L. Peptidoglycan Modifications Tune the Stability and Function of the Innate Immune Receptor Nod2. *J. Am. Chem. Soc.* **2015**, *137*, 6987–6990, doi:10.1021/jacs.5b01607.
5. Guzelj, S.; Nabergoj, S.; Gobec, M.; Pajk, S.; Klančič, V.; Slütter, B.; Frkanec, R.; Štimac, A.; Šket, P.; Plavec, J.; et al. Structural Fine-Tuning of Desmuramylpeptide NOD2 Agonists Defines Their In Vivo Adjuvant Activity. *J. Med. Chem.* **2021**, *64*, 7809–7838, doi:10.1021/acs.jmedchem.1c00644.
6. Gobec, M.; Tomašič, T.; Štimac, A.; Frkanec, R.; Trontelj, J.; Anderluh, M.; Mlinarič-Raščan, I.; Jakopin, Ž. Discovery of Nanomolar Desmuramylpeptide Agonists of the Innate Immune Receptor Nucleotide-Binding Oligomerization Domain-Containing Protein 2 (NOD2) Possessing Immunostimulatory Properties. *J. Med. Chem.* **2018**, *61*, 2707–2724, doi:10.1021/acs.jmedchem.7b01052.
7. Gobec, M.; Mlinarič-Raščan, I.; Dolenc, M.S.; Jakopin, Ž. Structural Requirements of Acylated Gly- l -Ala- d -Glu Analogs for Activation of the Innate Immune Receptor NOD2. *Eur. J. Med. Chem.* **2016**, *116*, 1–12, doi:10.1016/j.ejmech.2016.03.030.
